# Complete Genome Sequence of “*Candidatus* Syntrophocurvum alkaliphilum” Strain B(2M), Obtained from the Metagenome of a Salt-Tolerant Alkaliphilic Anaerobic Syntrophic Butyrate-Degrading Consortium

**DOI:** 10.1128/MRA.01511-19

**Published:** 2020-02-06

**Authors:** Andrey V. Mardanov, Dmitry Y. Sorokin, Alexey V. Beletsky, Nikolai V. Ravin

**Affiliations:** a Institute of Bioengineering, Research Center of Biotechnology, Russian Academy of Sciences, Moscow, Russia; b Winogradsky Institute of Microbiology, Research Center of Biotechnology, Russian Academy of Sciences, Moscow, Russia; c Department of Biotechnology, Delft University of Technology, Delft, The Netherlands; Indiana University, Bloomington

## Abstract

A highly salt-tolerant and alkaliphilic syntrophic consortium that degrades butyrate under sulfate-reducing conditions was purified from a hypersaline soda lake in southwest Siberia. Here, we present the complete genome sequence of the syntrophic primary butyrate degrader in order to understand the molecular mechanisms of interaction between consortium members.

## ANNOUNCEMENT

Syntrophy is a tightly coupled mutualistic cooperation between different organisms (1). Syntrophic communities functioning under haloalkaliphilic conditions and capable of degrading nonfermentable organic compounds under anaerobic conditions have been discovered only recently and are currently poorly characterized, although syntrophic processes in ecosystems such as soda lakes can be one of the most important mechanisms in the mineralization of organics (2, 3). Evidence of a significant presence of *Clostridia* (*Syntrophomonadales*) and Deltaproteobacteria (*Syntrophobacterales*) species known to be involved in syntrophic conversions of volatile fatty acids was also recently obtained in metagenomics studies of anaerobic sediments of hypersaline soda lakes (4, 5). The object of our research is a syntrophic consortium isolated from sediments from a hypersaline soda lake in southwest Siberia that is capable of growth on butyrate under sulfate-reducing conditions at extreme salinity and pH (3). To study the molecular mechanisms of anaerobic butyrate oxidation and syntrophic interactions, we sequenced the metagenome of this consortium and obtained the complete genome sequence of the primary syntrophic partner.

The syntrophic culture consisting mainly of a primary butyrate degrader, “*Candidatus* Syntrophocurvum alkaliphilum” strain B(2M), and its hydrogenotrophic sulfate-reducing partner, Desulfonatronovibrio magnus, (3) was grown in a sodium carbonate-based medium (2 M total Na^+^, pH 9.5) in the presence of 20 mM each sodium butyrate and sulfate. The total DNA was isolated using the PowerSoil DNA isolation kit (MoBio) according to the manufacturer’s protocols. Metagenomic DNA was sequenced using the Illumina platform. The shotgun genome library was prepared using the NEBNext Ultra II DNA library prep kit (New England BioLabs, USA). The sequencing of this library on an Illumina HiSeq 2500 instrument using HiSeq rapid SBS run v2 sequencing reagents generated 16,217,272 single-end 250-nucleotide (nt) reads. In addition, metagenomic DNA was sequenced on a MinION device (Oxford Nanopore, UK) using the ligation sequencing kit 1D and FLO-MIN106 cells. Nanopore sequencing generated 223,808 reads with an average length of 16,036 bp. Nanopore reads were *de novo* assembled using miniasm v.0.3 (6). The consensus sequence was corrected using Racon v.1.4.3 (7), Medaka v.0.10.0 (https://nanoporetech.github.io/medaka/), and two iterations of Pilon v.1.22 (8). Illumina reads were assembled using SPAdes v.3.13 (9); the corrected miniasm assembly was passed to SPAdes to order the assembly graph (–untrusted-contigs option). As a result, the complete circular genome of “*Ca.* Syntrophocurvum alkaliphilum” strain B(2M) was assembled; no plasmids were detected. Gene search and annotation were performed using the RAST server (10). The default settings were used for all software.

The length of the genome was 2,360,781 bp, with a G+C content of 32.5%. A total of 2,311 protein-coding genes were predicted. The completeness of this genome was estimated with CheckM v.1.05 (11) as 97.22%, with 1.06% possible redundancy. “*Ca.* Syntrophocurvum alkaliphilum” strain B(2M) was described as a member of a new genus-level lineage in the family *Syntrophomonadaceae* (*Firmicutes*) based on the 16S rRNA gene sequence (3). Taxonomic assignment of this genome to the Genome Taxonomy Database using the GTDB-Tk v.0.3.2 tool (12) confirmed that it belongs to this family. Genome analysis revealed genes for anaerobic butyrate oxidation (13, 14).

In summary, the complete genome sequence of “*Ca*. Syntrophocurvum alkaliphilum” strain B(2M), representing a novel candidate genus of *Syntrophomonadaceae*, was obtained. These genomic data will allow us to describe this lineage at the genomic level, as well as to understand syntrophic interactions in a butyrate-degrading consortium under haloalkalophilic conditions.

### Data availability.

The complete genome sequences of “*Ca.* Syntrophocurvum alkaliphilum” strain B(2M) were deposited in GenBank under the accession number CP046457. The raw sequences have been deposited in the Sequence Read Archive under the accession numbers SRR10662766 and SRR10673244. The version described in this paper is the first version (CP046457.1).
